# Making SharePoint^® ^Chemically Aware™

**DOI:** 10.1186/1758-2946-4-1

**Published:** 2012-01-12

**Authors:** Kartik Tallapragada, Joseph Chewning, David Kombo, Beverly Ludwick

**Affiliations:** 1 Targacept, Inc., 200 East First Street, Suite 300, Winston-Salem, NC 27101-4165, USA

## Abstract

**Background:**

The use of SharePoint^® ^collaboration software for content management has become a critical part of today's drug discovery process. SharePoint 2010 software has laid a foundation which enables researchers to collaborate and search on various contents. The amount of data generated during a transition of a single compound from preclinical discovery to commercialization can easily range in terabytes, thus there is a greater demand of a chemically aware search algorithm that supplements SharePoint which enables researchers to query for information in a more intuitive and effective way. Thus by supplementing SharePoint with *Chemically Aware*™ features provides a great value to the pharmaceutical and biotech companies and makes drug discovery more efficient. Using several tools we have integrated SharePoint with chemical, compound, and reaction databases, thereby improving the traditional search engine capability and enhancing the user experience.

**Results:**

This paper describes the implementation of a *Chemically Aware*™ system to supplement SharePoint. A Chemically Aware SharePoint (CASP) allows users to tag documents by drawing a structure and associating it with the related content. It also allows the user to search SharePoint software content and internal/external databases by carrying out substructure, similarity, SMILES, and IUPAC name searches. Building on traditional search, CASP takes SharePoint one step further by providing a intuitive GUI to the researchers to base their search on their knowledge of chemistry than textual search. CASP also provides a way to integrate with other systems, for example a researcher can perform a sub-structure search on pdf documents with embedded molecular entities.

**Conclusion:**

A *Chemically Aware*™ system supplementing SharePoint is a step towards making drug discovery process more efficient and also helps researchers to search for information in a more intuitive way. It also helps the researchers to find information which was once difficult to find by allowing one to tag documents with molecular entities and integrating with image recognition software to find information from pdf documents.

## Background

The amount of data generated during the transition of a single compound from preclinical stages to commercialization can easily range in Terabytes. So just imagine having a million compounds and the amount of data accumulated against them would be overwhelming. According to a survey [1], over 42 million biological test results were deposited in the PubChem database with 761,772 unique chemical structures. In the above scenario a traditional text search would fail to aggregate all the data; for example if one searched for varenicline, a compound launched by Pfizer as an aid to smoking cessation treatment [2], it would exclude the content pertaining to Chantix/Champix (trade names in the USA and Canada, respectively) and varenicline's IUPAC name (7,8,9,10-tetrahydro- 6,10-methano- 6*H*-pyrazino (2,3-h)(3) benzazepine).

Given the same example above, if one has content in SharePoint database referencing Chantix, varenicline, Champix, or the applicable IUPAC name and then searches on "Chantix" alone, SharePoint will return only the content which references Chantix, since it does not know that the other two are just synonyms of the same compound. Thus, we realized that SharePoint 2010 software is not aware of the variety of terms used in drug discovery to refer to the same thing. The other problem that surfaces is having a central location where a user can retrieve all the related information pertaining to a single compound. SharePoint 2010 software has the ability to connect to various databases (SQL Server, Oracle, DB2) [3], thereby enabling SharePoint software to not only query compound, reaction, and chemical inventory databases but also external databases by using web services and Accelrys Pipeline Pilot (Text Mining) capabilities [4].

Another interesting scenario where an enhancement to SharePoint is important is having a chemotype associated with carcinogenic adverse events and then wanting to see if there are any documents or compounds referencing that chemotype. CASP would allow one to perform a substructure search of such a chemotype and the results will return all the content containing that specific chemotype.

A *Chemically Aware*™ system not only saves time for searching but also helps in increasing the productivity of the work force. It is an invaluable business tool which helps in minimizing the effort and frustration to find a document but also enhances the productivity of a research and development group. Rick Rietz [5] correctly points out the scenario of finding information quickly when needed and how SharePoint's search helps the business to save on time and money, he also points out a quick scenario showing that how much time and money an organization would save by implementing SharePoint. Table 1 summarizes the below described formula using various scenarios.

**Table 1:** Different scenarios using the formula [5] to showcase approximately the amount of money saved by using a SharePoint search engine

Total Hours Spent on Searching/Day (minutes)	Total Number of Weeks(weeks)	Total Number of Employees(Employees)	Approximated Average Pay Rate($)	Total Money Saved by an Organization/Year($)
30	52	50	25	32,500
30	52	150	35	136,500
60	52	100	30	156,000
60	52	200	30	312,000

The numbers used in this table are arbitrary and may change from one organization to another.

The Total minutes spent per Day = m

The Total number of employees = e

Total Number of weeks in a year = y

Average pay rate = r

Total amount of Money Saved by an organization per year = $ r(m*e*y/60)

CASP can enhance many out-of-the-box collaboration features like discussion boards, blogs, etc. Commercially available SharePoint does not allow one to have chemical structure as a metadata column, but CASP allows one to tag documents (such as patents in a format not suitable for optical character recognition) and lists with two-dimensional chemical structures, which can then be found when performing a structure-based search. A chemist can then check if a structure is covered by a patent by doing a substructure search and retrieving a document that previously was not structure searchable.

In this paper, we address each of the above stated problems. We demonstrate how to supplement SharePoint features and integrate to make a *Chemically Aware*™ system.

## Requirements

Project Name: CASP

Operating System: Windows Server 2008 r2

Programming Language: C# and Perl

Other Requirements: SharePoint Server 2010, Accelrys^® ^Direct cartridge [4], Pipeline Pilot 7.5 [4], Accelrys JDraw 1.1.100.39 [4], IUPAC batch Naming 12 from ACD Labs [6] and Visual Studio 2010.

### Setup

CASP is set up after the standard installation of SharePoint software and is agnostic of the server farm configuration. Any successful SharePoint software implementation depends on how well the taxonomy is defined. To setup CASP, taxonomy is equally important for identifying common metadata, content and workflows within the enterprise, which can contain instances of some groups tagging the documents with smiles string, whereas other groups might tag the compounds with InChI key or IUPAC names.

In our study, we have used 2D structure representation, which encompasses all of the above metadata tags. Such a representation allows the users to visually identify the compound, unlike smile string or InChI key representation. In addition, for an internal SharePoint implementation within a pharmaceutical industry setting, most of the content can be tagged with a unique internal company compound ID. The following steps start the process for achieving a *Chemically Aware*™ system utilizing SharePoint. Additional files containing setup information and details is also provided at the end of the manuscript.

## Results

### a) How to Add a Structure Search Web Part

Step1. Take the Jar files from Accelrys JDraw and copy them into Program Files\Common Files\Microsoft Shared\Web Server Extensions\BIN folder.

Step2. Create a SharePoint Visual Web Part project using Visual Studio 2010 and name it, for example, *Structure Box*.

**Figure 1:**
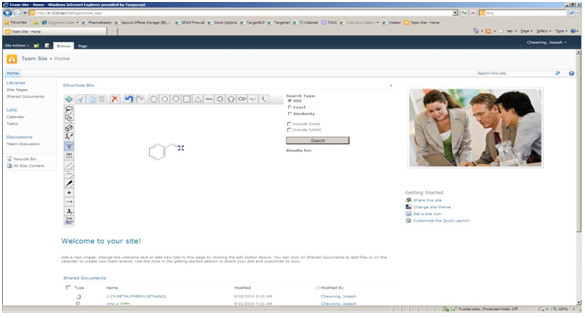
**Accelrys JDraw applet in a web part deployed on SharePoint**. The web part also has the exact, substructure or similarity search functionality built in.

Step3. Add an Ajax Update Panel and encompass the Jdraw Editor in a content template as described in Figure 1.

Step4. Take the molstring from the hidden value and query your internal database for compound ID, send the molstring to Pipeline Pilot to get the IUPAC name (powered by ACD Labs [6]), or take the molstring and query the SPStructure database table (refer to section (b) for more details) to find the documents by using the Document ID feature.

**Figure 2:**
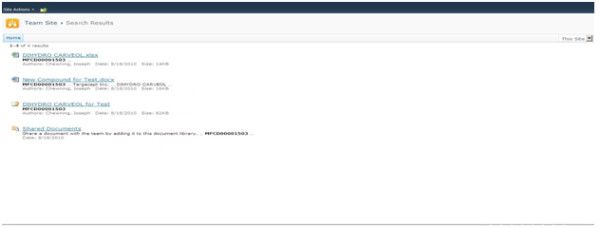
**Search results derived from a substructure search from the *structure box *web part**.

Step5. Based on the filter set by the user, display the results by sending the query to the search engine as shown in Figure 2.

Note that you can also enhance the Advanced Search Web Part in SharePoint by adding the Accelrys JDraw applet to the web part and search SharePoint content with both basic and advanced features. Please refer to additional file 1 for further setup information.

### b) Writing a Custom Crawl Feature to Achieve a *Chemically Aware*™ system utilizing SharePoint

Step1. Create a table called SPStructure in your internal structure database (for this project, we have created a table in our internal Accelrys Direct Database).

Step2. Apart from the molstring (ctab) column, add a column to store the DocumentID from SharePoint in the SPStructure table (DocumentID varchar2 (200 bytes)).

Step3. Create a blank SharePoint project in Visual Studio 2010.

Step4. Loop through all the documents and collect objects in a dictionary object (tested using Microsoft Word, Excel and PowerPoint documents).

Step5. Use the MDL Draw Renderer API to get the molstring, by copying the objects and pasting them to the Draw Renderer.

Step6. Save the molstring (ctab) and the Document ID in the SPStructure table.

Step7. Create a nightly job to automate the crawl process.

### c) Adding Structure as a Metadata Field to Content in SharePoint

Step1. Create an empty SharePoint project and add two class files, DrawField.cs and DrawControlField.cs. The DrawField.cs file should inherit from the base control class to add our custom structure field, and overwrite the createchildcontrol field to let the structure editor show up in the edit mode.

Step2. Add a CustomFieldControl.xml file and use the fieldtypeclass to reference the drawcontrol.dll, to render the drawn structure and display as metadata in SharePoint.

Step3. Deploy the application, map the \\Program Files\Common Files\Microsoft Shared\Web Server Extensions\14\TEMPLATE\xml folder in the 14-Hive and deploy the CustomFieldControl.xml to this folder.

Step4. To display the structure in SharePoint, we have used Accelrys Pipeline Pilot to get the related image for the drawn structure.

**Figure 3:**
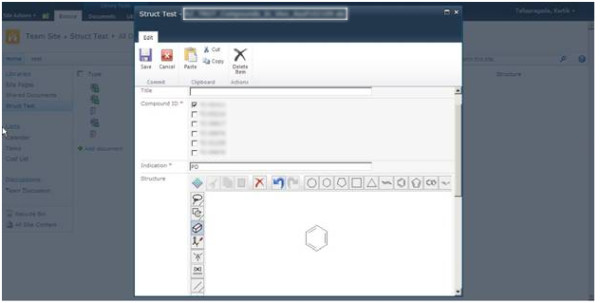
**Draw the chemical structure corresponding to the document using the Jdraw applet as shown above**.

**Figure 4:**
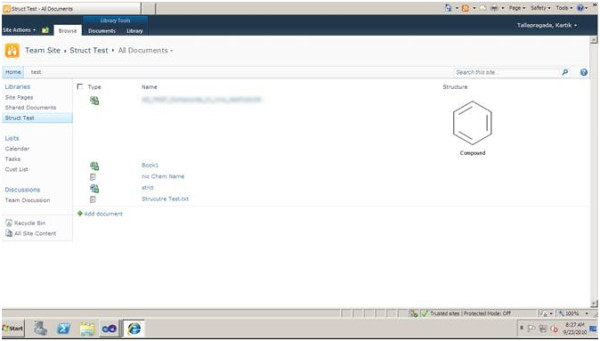
**CASP Storage of structure and its corresponding molstring as metadata**.

Step5. Test the application by clicking Edit Properties on a file and the Accelrys JDraw applet will be displayed as shown in Figure 3. Draw the related structure and save it. The structure will be displayed in SharePoint as a picture, as shown in Figure 4. The related molstring is saved in another hidden column.

### d) How do all the above steps contribute to the foundation for a *Chemically Aware*™ system utilizing SharePoint?

**Figure 5:**
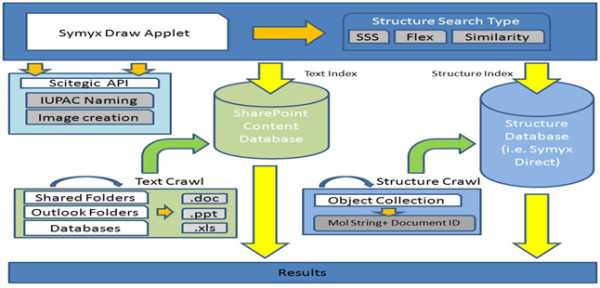
**CASP system architecture**. Orange arrow indicates internal process; yellow arrows indicate functional process, and green indicates nightly process.

CASP uses SharePoint software to run most of its processes, but to get the chemically rich features it uses tools like Accelrys JDraw and Pipeline Pilot to help render the chemical structure and help search documents with embedded chemical structures. Using the Accelrys Direct cartridge, one can perform various types of structure searches like Sub Structure similarity, Flex match, or exact structure similarity. One can also connect to the Pipeline Pilot protocol and retrieve information like IUPAC name, or any of the molecular properties in a SharePoint database list. The user interacts with CASP at the top level of the architecture as shown in Figure 5. The Accelrys JDraw applet is used for substructure, exact or similarity searches; these searches are processed by the Accelrys Direct cartridge running on Oracle. Other internal processes of the architecture include the Scitegic API, IUPAC Naming (ACD Labs) and custom Pipeline Pilot protocols which all together form the user interface. The second and third layers in the architecture are tightly coupled together and help in retrieving the results.

## Discussion

The text crawl is a native SharePoint software crawl feature which is done in order to find content by the search engine. CASP uses this feature not by customizing the crawl feature but by leveraging it to be *Chemically Aware*™. For example from the search UI, the user can draw a compound; send IUPAC names, smile string, or in-house compound IDs to the search engine. Since the content is crawled, the search results will find the data. The above text crawl solution might find 60% of the data in an enterprise, but if a compound of interest falls in the other 40%, it becomes very important to retrieve the data and show them in the search results. This led us to write a custom crawl feature which looks for embedded structures in documents and stores them in the Accelrys Direct database along with the Document ID. Document ID service is a new feature in SharePoint 2010 software which assigns a unique ID for each document. This is helpful in many ways in that we no longer have to worry about broken links or documents stored somewhere deep in a site collection [3]. This again ties back to the UI where the compound search first searches the structure database, retrieves the document ID and sends it to the search engine to show the resulting documents.

Although the custom crawl procedure helps us retrieve 80% of the documents, there are still 20% of the documents which are difficult for the search engine to find. Table 2 displays some of the searches performed by CASP using various search terms. Searches include structure (for ex nicotine, quinuclidine), SMILE strings, and basic text search terms.

**Table 2:** Several search terms were used to test the validity of CASP and the percentage of successful documents returned are shown in the table above

Search Term	Search Method	**Number of documents containing the search term**.	**Actual number of documents returned after search**.	**% documents retrieved**.
	Exact Similarity Search	1538	1359	88%
	Exact Similarity Search	384	280	72%
CCC1CCCCC1	Basic Text Search	234	207	88%
Nicotine	Basic Text Search	1538	1496 *	97%

Basic text search retrieved content containing nicotine but was unable to retrieve "non ocred" pdf documents, images of nicotine and pdb files.

One example is PDFs containing images for structures. These files cannot be indexed by the SharePoint text crawl since these are scanned images in a PDF file, nor can these files be indexed by our custom structure crawl since the compounds are not embedded objects but are images. There are solutions which would conduct a Chemical OCR on these types of files, but currently the precision is not optimal. So how does CASP handle these kinds of files? There are two ways to handle such files, as follows;

### a) Add Metadata to the Content using a CASP Structure Editor

Adding the chemical structure as metadata allows the custom crawl to index these files, thereby enabling these files to be structure searchable. Although this process of adding structure as metadata to each file will initially be very tedious, it will yield more accurate results than any Chemical OCR tool.

### b) Use Chemical OCR Capabilities

Another way to accomplish the task is to use a Chemical OCR component (OSRA) [7] to index the content. Below are the steps to accomplish this procedure.

1. Copy the Pipeline Pilot protocol for OSRA and point the "osra.bat" parameter to osra.bat script on Windows or "osra" executable on linux [7]

2. In the SharePoint custom crawl feature, add a method to send the PDF file as source to the protocol.

3. Read the output SD file, save the molstring and the Document ID to the SPStructure table in the structure database, so that if a user searches for a substructure the related PDF document will be found.

OSRA requires 300 dpi resolution for scanned documents and 72 dpi for images [8]. By adding a chemical OCR feature to the crawl process, it does delay the crawl time. Moreover, it's a CPU intensive process. Although this procedure does not yield 100% accurate results, it gives an idea as to how simple and powerful setting up CASP can be. Furthermore, this procedure can be integrated with a powerful chemical OCR tool which would enable a user to virtually find every electronic document in the enterprise based on the search query.

CASP provides a very powerful search tool which can eventually mine data accurately and efficiently at high speed. It is also important to mention that CASP is customizable. The fact that it is a platform built by leveraging in-house tools makes it customizable according to the need of any enterprise.

Finding data related to a query is probably the most important aspect of any document management system and has been a challenge for organizations. Although the search algorithm from Microsoft and the capabilities of a CASP system would narrow the path to finding your query related search results, there is still room for improvement. Examples include being able to query for a structure where the search results would let one know if the queried structure is covered by a specific type of patent (for example, a patent containing markush structures). Moreover, there is a possibility of having a SharePoint discussion board where chemists interact by drawing a structure, then modifying it as needed. This would be very useful for the enterprises which have overseas branches where a chemist in the US can discuss a structure and share ideas with colleagues abroad. Such a scenario certainly improves the drug discovery process since the chemist is not working in a vacuum because the ideas are being shared in real time using a collaboration platform.

## Conclusion

Setting up a *Chemically Aware ***™ **system in a SharePoint software environment lets an enterprise find content which previously would have been difficult to search or would have completely been "hidden" from out of the box text search queries. Leveraging a powerful collaboration tool like SharePoint software to create a *Chemically Aware*™ system definitely enriches chemists with knowledge from different sources (internal databases, external/public databases literature, and competitive intelligence). These techniques certainly improve the drug discovery process by enabling the chemist to not only explore other scaffolds, but also making it convenient to search different databases from a single portal. Chemical structures are represented in so many formats (smiles, IUPAC names, InChi, molstring, CML, common names), it is challenging for any chemical search engine to find all the results related to a drawn structure. CASP solves this problem by integrating SharePoint with other tools like Pipeline Pilot, Accelrys JDraw applet and ACD Labs to find the content related to the query. With the increase in chemical literature and the number of companies moving towards an enterprise solution for document management, the need for a *Chemically Aware*™ system becomes more important to speed up the discovery of innovative drugs capable of addressing many unmet medical needs.

## Methods

### SharePoint 2010

Microsoft SharePoint is enterprise class software that provides collaboration experience along with a strong, consistent, development platform that can be used to tailor the SharePoint experience to meet corporate and user needs [9]. SharePoint 2010 has an improved search algorithm for better matching and ranking capabilities to improve search results relevance across various types of content.

### Pipeline Pilot

Pipeline Pilot and its integrated set of applications address the modeling and simulation, informatics and scientific business intelligence needs of research and development organizations [10]. We have used Pipeline Pilot in this project to render the image for a molecular entity and also to run the OSRA protocol to capture the images and store them into the database.

### Accelrys JDraw

Accelrys JDraw is a lightweight, no-fee structure editor for web application development [11]. JDraw comprises of the UI framework of CASP, and the researchers are able to search for documents specific to a structure using the JDraw interface. JDraw helps one to cut and paste among other structure editor tools like Accelrys Draw, ISIS/Draw and ChemDraw. It also has the ability to edit the structure in place.

## Competing interests

The authors declare that they have no competing interests.

## Authors' contributions

KT and JC initially identified the need of a chemically aware system to supplement SharePoint 2010 to improve the drug discovery process by allowing the scientist to search for molecular entities using a single platform which would search various databases and documents and provide information to the scientist in a quick and timely fashion. KT has been responsible to design the UI and the source code to connect the various tool to SharePoint. DK and JC have been responsible for generating protocols using pipeline pilot to render images and also to save the chemical feature into the database. BL was responsible to setup the database connectivity framework to accommodate the storing of the chemical structure along with the document ID. KT has been the main author for the manuscript, while all authors have contributed by revising and further developing the content.

## Supplementary Material

Additional file 1 **Making SharePoint Chemically aware Source Code**. Using the source code from the above link and the steps described in the manuscript one can setup a chemically aware system. Please make sure to test this procedure in the test environment before deploying it into production. Click here for file
