# Supplementary material for: Making SharePoint® Chemically Aware™
Source: J Cheminform. 2012 Jan 12;4:1. doi: 10.1186/1758-2946-4-1 (PMC3275471; doi:10.1186/1758-2946-4-1)
Supplement: Additional file 1 — Making SharePoint Chemically aware Source Code. Using the source code from the above link and the steps described in the manuscript one can setup a chemically aware system. Please make sure to test this procedure in the test environment before deploying it into production. [file 1758-2946-4-1-S1.DOCX]

# How to setup the CASP Structure Editor using Symyx JDraw and SharePoint 2010.

Note to the readers. The source code below is written using C# and was developed using Visual Studio 2010. Please read the manuscript before implementing CASP. It is also recommended and best practice to implement and test the process in a QA environment and then implement in production. Also make sure to take a complete backup of the production environment before implementing CASP.

1. ***Source Code Part 1 for StructureBox.ascx***

<script type = “text/javascript”>

Function GetMolFileString(){

var applet = document.getElementbyID(“myJdrawApplet”);

};

<asp:UpdatePanel ID=”UpdatePanel1” runat=”server” UpdateMode=”Conditional”>

<ContentTemplate>

<applet code=”com.symyx.draw.JDrawEdition” name=”JdrawEdition” id=”myJdrawApplet”

height=”500” hspace = “0” vspace=”0” align=”middle” archive= ”/_layouts/ClientBin/jdrawApplet.jar, /_layouts/ClientBin/CSInline.jar,

/_layouts/ClientBin/jdrawcore.jar” style=”width:562px”>

1. *The DrawControl.cs inheriting from SPURL Field. Notice the initialization on the DrawControl in the FieldRenderingControl which enables the DrawControl to show up in the Edit Properties window.*

public override object GetFieldValue(string value)

{

If (String.IsNullOrEmpty(value))

return null;

Microsoft.SharePoint.SPFieldUrlValue DrawControlValue = new SPFieldUrlValue(value);

}

public override Microsoft.SharePoint.WebControls.BaseFieldControl FieldRenderingControl

{

[SharePointPermissions(SecurityAction.LinkDemand, ObjectModel = true)]

get

{

Microsoft.SharePoint.WebControls.BaseFieldControl DrawControl = new DrawControl();

DrawControl.FieldName = this.InternalName;

return DrawControl;

}

}

1. *Custom field to render the structure box in SharePoint, so that users can pick the field and assign it to content wherever applicable.*

<FieldTypes>

<FieldType>

<Field Name=”TypeName”>CustomWebField</Field>

<Field Name=”TypeDisplayName”>Custom Web Field</Field>

<Field Name=”TypeShortDescription”>Structure Field</Field>

<Field Name=”ParentType”>URL</Field>

<Field Name=”UserCreatable”>TRUE</Field>

<Field Name=”ShowInListCreate”>TRUE</Field>

<Field Name=”FieldTypeClass”>CASPProject.DrawControlField,CASPProject,Version=1.0.0.0,Culture = neutral, PublicKeyToken=b80b8a39110600f4</Field>

<RenderPattern Name=”PreviewDisplayPattern”>

<HTML><![CDATA[[“]]></HTML>

<Property Select = “DisplayName” HTMLEncode = “TRUE”/>

<HTML>”$Resources :core ,fldtypes011;</HTML>

</RenderPattern>

</FieldType>

</FieldTypes>
